# Operating room nurses’ lived experiences in the management of critical multiple and combined trauma: a qualitative study

**DOI:** 10.3389/fmed.2026.1858690

**Published:** 2026-06-01

**Authors:** Xulin Zhang, Suhui Li, Weidong Gong, Feng Lu, Panfeng Li, Jun Yin, Yuanzhi Li, Zhangyi Wang, Yan Wang

**Affiliations:** 1 Operating Room, The Second Affiliated Hospital of University of South China, Hengyang, Hunan, China; 2 Gastrointestinal Surgery/National Key Clinical Construction Specialist General Surgery, Clinical Medical Technology Demonstration Base for Emergency Treatment of Chest Pain in Hunan Province, The Affiliated Hengyang Hospital of Hunan Normal University & Hengyang Central Hospital, Hengyang, China

**Keywords:** critical multiple and combined trauma, multidisciplinary teamwork, occupational stress, operating room nurses, qualitative research

## Abstract

**Background:**

Critical multiple and combined trauma is severe, complex and rapidly progressive, posing great challenges in trauma care. Patient outcomes rely on prompt, efficient operating room teamwork. As key team members, nurses provide precise instrument support and emergency management while under heavy physical and psychological stress. Existing studies focus on clinical protocols, with little qualitative research on their real experiences, practical difficulties and needs. This study explores their lived experiences to optimize trauma care, relieve occupational stress, and improve multidisciplinary teamwork.

**Methods:**

This descriptive phenomenological study recruited 15 operating room nurses with ≥3 years of trauma nursing experience from a tertiary hospital in Hunan using purposive sampling with maximum variation. Semi-structured interviews were analyzed using Colaizzi’s seven-step method. Trustworthiness was ensured through investigator triangulation, member checking with five participants, maintenance of a detailed audit trail, reflexive journaling, and external expert review.

**Results:**

Four key themes emerged: professional challenges and emergency response, dual effects of teamwork, psychological stress and coping, and career development needs. Nurses managing critical multiple and combined traumas bear heavy technical and psychological burdens, seeking better critical care support and emergency decision-making skills. Despite efficient interdisciplinary collaboration being recognized as essential, communication conflicts, delayed staff support, and the emotional labor of absorbing others’ negative emotions were identified as persistent dilemmas. Nurses experienced anxiety and compassion fatigue, relieved by self-regulation and peer support, yet explicitly expressed unmet needs for professional psychological counseling, scenario-based simulation training, standardized procedures, and optimized staffing and emergency supplies.

**Conclusion:**

Enhancing trauma nursing for critical multiple and combined injuries requires targeted training, structured psychological support, optimized multidisciplinary collaboration, and systematic career development attention. These strategies, derived from nurses’ lived experiences, aim to improve both patient outcomes and professional wellbeing. Readers should assess transferability to their own settings.

## 1 Background

Trauma represents a major global public health challenge, responsible for approximately 8% of total deaths worldwide and the leading cause of mortality among people under 45 in developed countries (1). Multiple and combined traumas are the most severe and difficult-to-manage conditions in trauma care. Multiple trauma is defined as two or more anatomical injuries (classified by the AIS 9-region system) caused by a single mechanical force such as traffic accidents or falls from height (2). Severe multiple trauma is associated with a mortality rate of 20–70% without timely and effective treatment (3). Combined trauma results from two or more different injurious forces (thermal, blast, mechanical) acting simultaneously or sequentially, with at least one life-threatening injury, such as thermal compression injury or burn-blast injury (4). Its core characteristic is a synergistic effect: pathophysiological responses interact and amplify each other, leading to far greater severity than simple additive injuries and significantly higher treatment complexity than conventional trauma (5). Consequently, such injuries often involve multiple organs and systems, with rapid disease progression, complex pathophysiological mechanisms, and high complication rates. The overall mortality ranges from 30 to 50%, making these injuries a key challenge in trauma care that imposes a substantial burden on health care systems worldwide (6). In recent years, the incidence of trauma has risen steadily in China. Although regional trauma centers have been established successively, a considerable shortage of specialized trauma nurses remains. High complication rates attributable to inadequate nursing coordination in the management of multiple and combined traumas further underscore the urgent need for specialized trauma nursing (7). As a life-saving intervention for patients with multiple and combined traumas, emergency trauma surgery differs fundamentally from elective surgery. Trauma procedures are unplanned rescues involving hemodynamically unstable patients, multiple concurrent injuries, and high hemorrhagic risks. This high-stress, unpredictable setting requires rapid assessment, intervention, and seamless teamwork, imposing exceptionally high professional demands on operating room nurses. A recent scoping review of 36 studies by Tjugum et al. (8) specifically mapped the psychosocial factors that influence occupational stress in operating room nurses, identifying workload, interprofessional challenges, and moral distress as primary contributors to the demanding nature of this environment. As key players in trauma care, nurses serve as both surgical workflow executors and critical emergency collaboration hubs: preoperatively, they quickly prepare instruments, verify equipment, and coordinate the team; intraoperatively, they assist precisely, respond to sudden clinical changes, and their timeliness and accuracy directly impact patient treatment outcomes. Efficient interdisciplinary team communication and coordination are essential for complex trauma rescue, and smooth cooperation among medical staff can effectively guarantee the quality of trauma care and improve patient prognosis (9). Beyond technical tasks, nurses act as a communication bridge, conveying critical information among the surgical team, patients, and families. Surendran et al. (10), in their qualitative study of emergency nurses, highlighted that excessive cognitive and emotional demands in high-pressure, resource-limited environments can compromise both clinician wellbeing and patient care delivery. This finding is directly relevant to the trauma operating room context, where nurses face similar dual pressures of clinical precision and emotional labor. Heavy and diverse workloads expose operating room nurses to sustained physical and psychological stress. Psychologically, repeated exposure to traumatic scenes, critical illness, and poor outcomes place nurses at high risk of secondary traumatic stress (STS) and vicarious trauma (VT). Studies report the prevalence of secondary traumatic stress among trauma nurses ranges from 25 to 45%, with the prevalence of vicarious trauma reaching 20–50% among nurses in emergency and trauma-related settings (11). Caulfield et al. (12), in their integrative review, synthesized evidence on the factors preceding occupational distress among emergency nurses, identifying individual, organizational, and occupational sources that contribute to burnout, compassion fatigue, and secondary traumatic stress. These factors closely parallel those reported by trauma operating room nurses in our study. However, most existing studies have focused on emergency department settings, with limited qualitative work specifically addressing the intraoperative trauma context. In recent years, trauma nursing research has increasingly focused on the real psychological experiences and occupational dilemmas of nurses during clinical rescue, such as stress responses among emergency nurses responding to mass blast injuries (13). However, most relevant studies have centered on the work status of nurses in routine elective surgery, or have been limited to quantitative analyses of single technical topics. Regarding training systems, most existing programs focus on general nursing skills or broad curriculum rollouts (14). Despite the widely recognized importance of trauma nursing, notable gaps remain. Existing training programs are largely restricted to isolated pre-hospital or in-hospital steps, lacking a standardized, immersive system for complex injuries (14). Furthermore, while studies have examined psychological stress among operating room nurses during resuscitation (7), few qualitative works have deeply explored OR nurses’ real-time psychological experiences and professional needs specifically in the context of critical multiple and combined trauma surgery. A concept analysis by Baik et al. (15) identified six key attributes of trauma nursing competency—rapid initial assessments considering injury mechanisms, priority determinations based on urgency and severity, clinical knowledge of trauma nursing, skills of trauma nursing, interprofessional teamwork, and emotional care—providing a comprehensive framework that our study extends into the lived experience of the operating room context. These gaps leave existing training systems, psychological support strategies, and teamwork protocols poorly targeted and ineffective, failing to meet the practical clinical demands of critical multiple and combined trauma management.

## 2 Objective and research question

The overarching research question guiding this phenomenological inquiry was: “What are the lived experiences of operating room nurses in managing critical multiple and combined trauma patients, and what factors shape these experiences in the high-stakes surgical environment?” This main question was further explored through four sub-questions:

1. What are the real-time psychological experiences and perceptions of operating room nurses during coordination for such surgeries?
2. How do contextual factors influence their trauma response, teamwork, and professional development in this context?
3. How does surgical coordination affect nurses’ sense of professional mission, emotional regulation capacity, and professional competence?
4. What specific dilemmas and support needs do these nurses face?

This study adopts a qualitative phenomenological approach, aiming to describe and interpret, rather than test pre-defined hypotheses. Underpinning this inquiry are the phenomenological presuppositions that operating room nurses’ experiences in trauma care are complex, multi-dimensional, and shaped by the high-acuity clinical context; that these experiences encompass professional, psychological, and interpersonal dimensions; and that their meaning can be uncovered through systematic interpretation of narrative data. The study’s findings will fill the research gap regarding OR nurses’ specific experiences in coordinating for multiple and combined trauma surgery, enrich qualitative research outputs in the trauma nursing field, and provide a scientific basis for optimizing trauma surgical coordination training programs, building targeted psychological support systems, improving teamwork efficiency, and enhancing overall nursing quality.

## 3 Methods

### 3.1 Study design

This study adopted a qualitative research design. In line with the core principle of purposive sampling in qualitative research—focusing on the research core and selecting typical, representative samples (16)—we selected participants using the maximum variation sampling strategy proposed by Patton (17). We collected data via semi-structured in-depth interviews, determined the sample size per the data saturation principle, and aimed to deeply explore operating room nurses’ real experiences during collaborative care for critical multiple and combined traumas.

### 3.2 Participants and sampling

Using purposive sampling, we recruited operating room nurses from a tertiary A hospital in Hunan Province as study participants. As a regional trauma center with a well-established trauma care system, the hospital performs no fewer than 500 trauma surgeries annually across orthopedics, thoracoabdominal surgery, neurosurgery, and other disciplines, ensuring the representativeness of the sample. Following the maximum variation sampling strategy by Patton (17), we recruited heterogeneous participants across multiple dimensions: age (25–30 years, 31–40 years, >40 years), education level (associate degree, bachelor’s degree), work experience (3–5 years, 6–10 years, >10 years), professional title (Nurse, Practicing Nurse, Nurse-in-charge), and trauma surgery coordination specialty (orthopedic trauma, thoracoabdominal trauma, multiple trauma). This approach allowed us to fully capture the real experiences of nurses with different profiles, comprehensively reflecting the diversity and complexity of the research phenomenon. Inclusion criteria were as follows:

1. Holding a valid nursing practice license, with ≥3 years of continuous working experience in the operating room;
2. Having trauma surgery nursing experience, with independent coordination of ≥10 cases of multiple or combined trauma surgeries in the past 12 months;
3. Voluntarily enrolling in the study, with clear communication ability and capacity to accurately describe personal experiences.

Exclusion criteria were as follows:

1. Not participating in trauma surgery coordination in the past 12 months due to off-site training, long-term sick leave, or job departure;
2. Only assisting in trauma surgery coordination, with no independent coordination experience;
3. Voluntarily withdrawing from the study or being unable to complete the interview during the research process. We determined the sample size per the core principles of qualitative research outlined by Guest et al. (18). Data saturation, a key criterion for assessing qualitative study quality and justifying sample size (19), was applied: we stopped sampling when no new themes emerged from three consecutive interviews. Ultimately, 15 participants were included, coded P01 to P15, and their demographic characteristics are presented in Table 1.

**Table 1:** General information of interviewees (*n* = 15).

Code	Age (years)	Work experience (years)	Education	Professional title	Marital status	Trauma surgery specialty
P01	32	12	Bachelor’s degree	Senior nurse	Married	General surgery
P02	29	8	Bachelor’s degree	Staff nurse	Married	General surgery
P03	26	4	College degree	Registered nurse	Unmarried	Orthopedic trauma
P04	30	8	Bachelor’s degree	Staff nurse	Unmarried	General surgery
P05	35	13	Bachelor’s degree	Senior nurse	Married	Head and neck surgery
P06	30	7	Bachelor’s degree	Staff nurse	Married	Orthopedic trauma
P07	40	15	Bachelor’s degree	Senior nurse	Married	Urology
P08	28	7	Bachelor’s degree	Staff nurse	Married	General surgery
P09	36	15	Bachelor’s degree	Senior nurse	Married	Head and neck surgery
P10	32	9	Bachelor’s degree	Senior nurse	Married	Orthopedic trauma
P11	40	18	Bachelor’s degree	Senior nurse	Married	General surgery
P12	32	12	Bachelor’s degree	Senior nurse	Married	General surgery
P13	35	14	Bachelor’s degree	Senior nurse	Unmarried	Orthopedic trauma
P14	36	14	Bachelor’s degree	Senior nurse	Married	General surgery
P15	35	13	Bachelor’s degree	Senior nurse	Married	General surgery

### 3.3 Ethical approval

This study was approved by the Ethics Committee of the Second Affiliated Hospital of University of South China (Ethical Approval No. 2026017), and all procedures strictly followed the Declaration of Helsinki and relevant ethical norms.

After obtaining permission from the hospital management and the department’s head nurse, we communicated with and recruited potential participants. All participants signed written informed consent prior to the study: we fully informed them of the research purpose, procedures, risks and benefits, and explicitly clarified that they could withdraw unconditionally at any time without any negative consequences.

All participants were anonymized, with real names replaced by codes P01–P15. Raw audio recordings and transcripts were encrypted and stored on the hospital’s dedicated server, accessible only to authorized research team members. If participants presented intense emotional reactions during interviews, we immediately paused the process, and a counselor from the research team provided instant psychological support; we only resumed after the participant’s emotional state stabilized. After the study, we provided participants with preliminary results to safeguard their right to know.

### 3.4 Data collection

#### 3.4.1 Interview guide development

As the researchers and participants were colleagues from the same department, daily work interactions had already built basic communication and trust, which helped reduce social desirability bias in interviews and improve the authenticity of raw data.

Guided by the study objectives, we developed a semi-structured interview guide after consulting clinical experts. The core questions covered: core tasks of trauma surgery coordination, practical experiences in coordinating typical multiple and combined trauma surgeries, main difficulties and coping strategies, specific manifestations of occupational stress and adjustment methods, key nodes in professional growth, and suggestions for improving trauma nursing training systems and work environments. Each core question was supplemented with 3 to 5 probing directions (see Table 2 for details). We used facilitative questioning techniques during interviews to ensure data saturation.

**Table 2:** Semi-structured interview guide.

Question code	Core question	Probing directions
1	Core tasks of trauma surgery coordination	1. Daily workflow; 2. Frequency of participating in multiple/combined trauma surgeries; 3. Core difficulties of the work; 4. Differences from elective surgery coordination.
2	Impressive experiences in coordinating multiple combined trauma surgeries	1. Patient condition characteristics; 2. Key coordination links; 3. Your emotional experience at that time; 4. Successes and regrets of the coordination; 5. Impacts of this experience.
3	Difficulties and challenges in surgery coordination	1. Specific skill-related difficulties; 2. Problems in teamwork; 3. Specific solutions you adopted; 4. Unresolved dilemmas; 5. Expected solutions.
4	Sources of occupational stress and coping strategies	1. Main sources of pressure; 2. Common self-adjustment methods; 3. Adjustment effects; 4. Expected external support; 5. Whether you had burnout intentions due to pressure.
5	Reflections on professional growth	1. Personal ability improvements from trauma surgery participation; 2. Manifestations of professional sense of value; 3. Suggestions for new nurses; 4. Key events in your own growth.
6	Suggestions for training and work environment	1. Deficiencies of current training; 2. Expected training content and format; 3. Specific demands for work environment optimization; 4. Suggestions for collaboration mechanism improvement; 5. Suggestions for staffing and resource allocation.

#### 3.4.2 Data collection implementation

We collected data via semi-structured one-on-one in-depth interviews between January 2024 and July 2025. All interviews were conducted in the hospital’s independent interview room during participants’ non-working hours (lunch break or after work) to avoid external interference. Each interview lasted 30–45 min. Most participants completed the process in one session, and those who interrupted for special reasons rescheduled a second one.

Prior to interviews, we informed participants of the research purpose, data use, and confidentiality commitment—real names were replaced with anonymous codes P01–P15—and obtained written informed consent. We used digital voice recorders to create dual backups of all recordings, and documented participants’ emotional reactions and non-verbal behaviors (such as silence, body movements) during the process.

All recordings were transcribed within 24 h after interviews. We analyzed the data using Colaizzi’s 7-step analysis method, and conducted member checking with 5 participants to verify the accuracy of the transcripts.

### 3.5 Data analysis methods

All interview recordings were independently transcribed into text by two researchers trained in qualitative research within 24 h of completion, followed by cross-verification to ensure data accuracy. Non-verbal cues (e.g., pauses, tone variations, and body language) documented during interviews were collated synchronously. For systematic data reduction and analysis, Colaizzi’s seven-step method was employed, with specific procedures as follows:

1. *Data immersion*: Each transcript was read repeatedly (at least 3 times) by two researchers independently to achieve full immersion in the data, capturing the core content, emotional tone, and key topics. Preliminary coding memos were developed concurrently.
2. *Extracting significant statements*: Guided by the core research question, we systematically extracted meaningful statements directly related to the topic and reflecting the essence of participants’ experiences, while excluding irrelevant and repetitive content.
3. *Formulating meanings*: Each meaningful statement was analyzed and assigned an initial code that precisely captured its core implication (e.g., “I felt extremely pressed for time, racing against time every second” was coded as “racing against time”; “I kept worrying I might do something wrong” was coded as “fear of error”).
4. *Clustering themes*: Using constant comparative analysis, initial codes with similar implications and shared experiences were categorized into logically coherent subthemes. The three-person research team conducted iterative discussions to reach consensus on all coding decisions.
5. *Developing exhaustive description*: Subthemes were further integrated and refined to explore their internal relationships, from which four core themes reflecting the full nature of the phenomenon were derived.
6. *Identifying the fundamental structure*: Core themes and subthemes were illustrated in detail with representative quotations from original data to ensure consistency with participants’ actual experiences.
7. *Member checking*: Five participants (33% of the sample) were invited to provide feedback on the analysis results to verify consistency between the themes and their actual experiences, achieving an 86% consistency rate. Two qualitative research experts also reviewed the theme extraction and result presentation.

NVivo 15.0 was used throughout to assist coding, word frequency analysis, coding matrices, and node relationship mapping, ensuring a systematic and transparent analytic process.

### 3.6 Trustworthiness

Methodological rigor was ensured through the application of the trustworthiness criteria established by Lincoln and Guba (20). Credibility was established through three strategies. First, investigator triangulation: two researchers jointly coded all transcripts through an iterative consensus process. Specifically, they read each transcript together, discussed each meaningful statement, and resolved any disagreements through immediate discussion, reaching 100% agreement on all codes. A third researcher then reviewed the final coding structure to provide an additional layer of validation. Second, prolonged engagement with the trauma operating room context enabled a nuanced understanding of the clinical environment. Third, member checking was conducted with five participants (33% of the sample), who reviewed and confirmed the accuracy of the themes with an 86% consistency rate, which is considered acceptable in qualitative research according to Lincoln and Guba’s (20) trustworthiness criteria, as it indicates substantial agreement while allowing for individual interpretive variation. The two discrepancies (14%) primarily involved the wording of one sub-theme label; these were discussed with the participants, and a revised wording acceptable to all five was incorporated into the final theme structure.

Transferability was facilitated through the provision of thick description in the Results section, including detailed participant quotations and contextual information about the research setting and sample characteristics.

Dependability was achieved by maintaining a comprehensive audit trail throughout the study, documenting all research decisions, methodological notes, data collection procedures, coding frameworks, and theme development processes. The audit trail is available from the corresponding author upon reasonable request.

Confirmability was ensured through two approaches. All researchers maintained reflexive journals throughout the data collection and analysis process to bracket personal assumptions, document emotional reactions, and minimize potential bias. Additionally, two external experts in qualitative research reviewed the study design, interview guide, and final theme structure to confirm that the findings were grounded in the data and not merely researcher constructs. These procedures collectively ensured that the findings met the Consolidated Criteria for Reporting Qualitative Research (COREQ) guidelines and accurately reflected the participants’ authentic experiences. The researchers and participants are colleagues from the same operating room department, which may increase the risk of social desirability bias (participants might present their experiences more positively). To mitigate this, we: (a) before each interview, explicitly told participants there were no “right” or “wrong” answers and that honest negative experiences were equally valuable; (b) assured participants that all data would be anonymized and would not be shared with any supervisor; (c) during analysis, actively coded for disconfirming instances (e.g., reports of conflict, emotional distress, unexpressed suffering) to counterbalance any positivity bias.

## 4 Results

Data were collected through in-depth interviews with 15 operating room nurses. Using Colaizzi’s seven-step approach, transcripts were systematically analyzed. Ultimately, four core themes and eight sub-themes were identified. All themes had clear connotations and distinct category boundaries, with each sub-theme supported by representative interview quotes. The corresponding relationships between core themes, sub-themes, and typical quotes are detailed in Table 3.

**Table 3:** Hierarchical structure of research themes and summary of typical quotations.

Core theme	Subtheme	Representative quote
Theme I: Professional challenges and emergency response	1.1 The rigorous challenge of complex multiple and combined trauma surgery	P15: “There was this patient with really severe injuries from a car accident. At that time, we did three surgeries on him all at the same time: craniotomy, thoracotomy, and laparotomy. His spleen ruptured, and his brain injury was also really severe. It was just like running two operating rooms at the same time, and the patient was bleeding like crazy the whole time.” P13: “I got called for an emergency surgery late one night. It was a combined trauma case. We did chest and foot surgery at the exact same time. The biggest challenge was, first, the patient was just so, so critical. And on top of that, we were doing two separate surgeries on this single patient all at the same time. And the scrub nurse only covered the chest part. So I had to handle the instruments for the leg surgery, plus my regular circulator work. Also, since the patient was in such bad shape, we had to do blood transfusions and all that, and assist the anesthesiologist too. Basically, I was juggling so many different duties at once just to get through this one case, it was so stressful.
	1.2 Core requirements for emergency response capability	P03: Because the surgeries were carried out together, I think we needed to pay more attention and be more careful when it comes to item counting and aseptic principles. Because the trauma contaminated the patient’s abdomen, and craniotomy has much higher sterile requirements. Also, we had to keep track of the doctors’ movements at all times during the operation, do not just throw our surgical instruments and gauze around randomly, to avoid mixing up the instruments and supplies for the two sites, which would lead to cross-infection between the two parts. P11: In this operation, I was the scrub nurse. This patient did not just have multiple fractures, he also had a ruptured spleen and liver injury. Right after the surgery started, the patient started hemorrhaging badly, his blood pressure dropped like a rock. And the atmosphere just got super tense all of a sudden. Once the doctors found the bleeding point, they needed to stop the bleeding right away. At that point, I had to get the hemostatic gauze and surgical instruments to them as fast as I could. I think the biggest challenge through the whole thing was staying super focused, passing the doctors exactly what they needed as fast as possible, because at a time like that, you really feel like the patient’s life is right in your hands.
Theme II. Value and dilemmas of team collaboration	2.1 Key supporting role of efficient collaboration	P05: I feel like trauma resus is totally a race against the clock. Everyone has their clear roles, but we all pitch in for each other too. Like, one person passes the instruments, one handles blood transfusions and IVs, and another monitors the vitals. When we click like that, it really shaves a bunch of time off the surgery, and makes it way more likely we can save the patient.” P11: I think this spirit of teamwork is really very important. Just like this patient with severe trauma caused by a car accident who needed surgeries on the brain, chest and abdomen at the same time. We had five medical staff in total, everyone performed their own duties, two instrument nurses and three circulatory nurses, responsible for blood transfusion, assisting the operation and various other tasks. Everyone stuck to their posts and cooperated closely. I think this efficient teamwork is the only way to ensure the smooth progress of the rescue. Without such excellent teamwork, we could never have completed such a complicated and high-risk surgery. P09: “The importance of teamwork is reflected in many aspects. First, it can improve surgical efficiency and success rate. Trauma surgeries are often complicated, and time is very tight. I think close cooperation between team members can optimize the surgical process, reduce unnecessary waiting and duplicate work. Also, through teamwork, we can find and correct potential errors in a timely manner, and avoid the occurrence of medical accidents. At the same time, when facing unexpected situations, team members can respond together, to protect the patient’s life safety to the greatest extent.”
	2.2 Practical dilemmas in collaboration	P04: “It’s just because everyone is in this high-pressure environment, sometimes the doctors just get really anxious. The anesthesiologist thinks his anesthesiology work is urgent, the surgeons think the surgical work is urgent, and you think your own work is urgent, then there will be conflicts and contradictions.” P05: “If it’s a newly hired anesthesiologist who lacks experience, based on my past experience, I will take the initiative to ask him if you need to prepare this, or if you need me to get this ready for you. If I really feel that this might be a bit beyond his ability, I might suggest to him if it’s necessary to call the second-line senior staff over, and so on.” P14: “When we are about to move to the next step, there’s something you cannot predict, you do not know it’s coming, and they ask you to do this thing. You will get anxious, and flustered. Because you will think, why did not I think of this in advance, why did the doctor have to remind me, and now I’m not ready all of a sudden.”
Theme III. Psychological stress and coping strategies	3.1 Psychological reactions in high-pressure environments	P12: “When facing patients with severe and complex conditions, I will naturally feel a lot of psychological pressure, very anxious and nervous, worried that I will not cooperate well in the surgery, the surgery will fail, or the patient will have a poor prognosis. Also, critical trauma patients’ conditions change extremely fast, we barely have time to get ready. We have to deploy all the instruments and supplies urgently right away, and at the same time worry that wasting this time will affect the patient’s treatment. This situation makes me extremely anxious. Sometimes when I see how severe the patient’s trauma is, I even feel scared, and even have compassion fatigue.” P14: “Right, because when receiving critical patients with multiple injuries and polytrauma, after all, we are saving lives, the whole rescue process is a race against time. The supplies preparation is also very complicated, we have to deploy all kinds of surgical instruments urgently, get the crash cart and all kinds of rescue supplies fully ready. In this situation, you will unconsciously get super tense, worried that the instruments and supplies will not be in place in time, delaying the critical time for the patient’s rescue. P10: “Usually, these patients often have complicated injuries, the surgical process is full of uncertainty and risks, a little negligence may affect the patient’s life safety. Also, the tense atmosphere in the operating room, and the anxious eyes of the family members, all make us feel that the responsibility is huge. We have to stay highly focused and alert at all times, make sure every operation is accurate. Plus, the surgery takes a long time, the work intensity is high, and we will feel tired both physically and mentally. But even so, we cannot relax even a little bit, because the patient’s life is right in our hands. So, the pressure is really very big.”
	3.2 Self-adjustment and demand for external support	P12: “How I deal with stress. Mostly, it’s just building up my professional skills. I do that by learning new stuff and going to training to get better at my professional knowledge and skills. Also, I work on getting my head right too. Like deep breathing relaxation exercises, and positive self-talk, to calm my nerves and keep a good headspace. Besides that, I talk things through with my team members. We share the stress and our experiences, support and cheer each other on, so we can face the hard parts and challenges during surgery together.” P01: “First of all, I will make preparations. We have a trauma center group. If I am on standby this week, I will keep paying attention once TTA is activated in the group. For such patients, I will keep following the examination results sent in the group, take the initiative to communicate with doctors about surgical plans before the operation, and make full preparations to relieve pressure. At the same time, I will get ready for possible accidents during the operation. After the operation, I will communicate and share with colleagues, review deficiencies in the cooperation process. But I still hope the hospital can provide professional psychological counseling services.” P07: “I think we need more staff. Patients with multiple injuries and combined injuries are in critical condition with rapid changes, so the staffing ratio must be different from that for elective surgeries. We also need more emergency surgical supplies, such as warming blankets, blood and infusion warming and pressurizing devices, so that there are enough for two simultaneous surgeries. In addition, we have a trauma center group. When on standby, I will keep an eye on the examination results shared in the group, communicate with doctors in a timely manner, and make preparations in advance to prevent intraoperative accidents, which can reduce panic.”
Theme IV. Professional growth and support needs	4.1. Enhancement of professional value	P01: “Personally, I think participating in the rescue of such patients many times has greatly exercised my psychological quality and emergency response ability. I can make correct responses quickly in such situations, and I feel quite proud. It has also strengthened effective communication with the team. From a professional perspective, my professional knowledge and skills have definitely improved. Through repeatedly participating in the treatment of multiple injuries and combined injuries, I have learned many treatment methods and surgical techniques for complex injuries. I can even offer some suggestions and guidance to young doctors during ordinary simple surgeries, thus gaining a sense of accomplishment from recognition. This also makes me believe that the mission of this profession is absolutely worth pursuing.” P09: “I feel these experiences have exerted a profound impact on my personal and professional development. First, I feel they have strengthened my adaptability and teamwork skills, allowing me to face various complex surgeries with greater confidence and composure. Second, I feel I have gained a deeper understanding of the importance of medical humanistic care. Last, I feel a strong sense of accomplishment to help critically ill patients regain their lives, and I am even more convinced that my work is meaningful.”
	4.2. Needs for improvement in training and work environment	P09: “I feel that teamwork among us could be further strengthened. Although our current cooperation is fairly good, we sometimes encounter problems such as delayed information transmission or misunderstanding. It would be even better if we could carry out more simulation drills or establish a more efficient information exchange platform. Regarding equipment, we sometimes face emergencies during operations that require special equipment or consumables. If these could be prepared in advance or there was a rapid access mechanism, surgical efficiency would definitely be greatly improved.” P02: “I have been working in the trauma operating room for quite some time, and we have developed certain procedures. These procedures can be documented in paper form so that new employees and less experienced staff can follow them when encountering such problems and avoid feeling flustered. P11: I think, sometimes, doing these kinds of surgeries is really so stressful. Sometimes I just feel really tired. Sometimes I might get scolded by doctors in urgent situations. And then sometimes I’m afraid I cannot handle the patient’s condition, so I get really anxious. If there were specialized psychological counseling, it would probably be a huge benefit to us.

### 4.1 Professional challenges and emergency response

#### 4.1.1 The rigorous challenge of complex multiple and combined trauma surgery

The care of patients with multiple or complex trauma, characterized by concurrent multi-site and multi-system injuries, imposes a unique and extreme demand on operating room nurses’ professional knowledge and clinical skills. This creates a clinical environment where nurses must navigate competing priorities in a race against time. One nurse vividly captured the profound weight of this responsibility and the intensity of focus it demands: P01: “The surgery had just started when the patient began to hemorrhage violently, and their blood pressure dropped sharply... The atmosphere immediately became extremely tense... I had to hand over hemostatic gauze and surgical instruments at the fastest possible speed. I think the biggest challenge of the whole process was having to concentrate at a very high level, to pass on to the surgeon what they needed as quickly as possible, because in moments like this, you really feel the patient’s life is in your hands.” This sense of holding a life in one’s hands is further complicated by the technical demands of concurrent procedures. For patients undergoing simultaneous multi-cavity surgeries, the risk of cross-contamination becomes a central, stress-inducing concern, requiring meticulous attention to detail amidst the urgency: P03: “Because these surgeries are performed concurrently, I think we need to pay even more attention to our instrument counts and sterile principles... The trauma has contaminated the patient’s abdominal cavity, but the simultaneously performed craniotomy has much stricter asepsis requirements... We can’t casually place surgical instruments and gauze, to avoid mixing up the items from the two surgical sites, which could cause cross-infection between them.”

P03’s account reveals a critical dimension of technical competence in trauma care that transcends speed alone: it is about managing the contradiction between the contamination of traumatic wounds and the imperative of multi-site sterility. This dual cognitive load—responding to hemorrhage urgency while concurrently upholding infection control—represents a balance not easily captured in routine training. In the most severe situations, this professional challenge culminates in an all-encompassing demand on the nurse’s perception: P15: “The patient had a very severe car accident injury; we were performing a craniotomy, thoracotomy, and laparotomy simultaneously... The biggest challenge was having to ‘keep your eyes on everything and your ears open in all directions’... The patient was hemorrhaging massively throughout.” The colloquial phrase “eyes on everything, ears in all directions” powerfully encapsulates the hyper-vigilance required of nurses in the trauma operating room. This state of diffuse attention—monitoring multiple surgical fields, tracking instruments, and anticipating the needs of surgeons and anesthesiologists simultaneously—represents a distinct form of cognitive and emotional load that defines the trauma nursing experience.

#### 4.1.2 Core requirements for emergency response capability

Trauma surgery is characterized by urgency and unpredictability, requiring operating room nurses to possess strong rapid response capabilities. When emergencies arise, nurses must quickly prioritize tasks and accurately anticipate surgeons’ needs. Meanwhile, they must strictly adhere to aseptic principles and surgical count protocols to prevent medical errors resulting from hasty emergency operations. Multiple participants noted that operational precision and process standardization under emergency conditions are the core guarantees of surgical safety. Participant P03 stated: “When abdominal surgery and craniotomy are performed concurrently, we must strictly segregate the instruments and supplies for the two sites to prevent cross-infection from mix-ups. Meanwhile, we need to coordinate with surgeons to rapidly complete hemostasis, blood transfusion, and other urgent tasks. This places extremely high demands on our response speed and operational precision.” Participant P12 added: “During emergency resuscitation, we not only need to act fast, but also prepare potential required instruments and supplies ahead of time. For instance, we need to prepare hemostatic materials, blood products, and resuscitation equipment in advance for cases of massive hemorrhage, so that we can keep up with the surgeons’ resuscitation pace.” Beyond immediate response, anticipatory preparation emerged as a defining feature of expert practice. A more experienced nurse described this capability as a cultivated professional instinct: P05: “Once you’ve accumulated enough work experience, you can absolutely take the initiative. Before the doctor even opens their mouth, we can already know what they might want next, and I’ll have it ready in advance. That’s a professional capability. Practice makes perfect—the more you do it, the faster your reaction becomes.” However, this level of mastery is not inherent; it develops through repeated exposure. A less experienced nurse highlighted the challenges of unfamiliarity: P06: “You need to keep up with the doctor’s rhythm at every step. You need agility and quick reactions. But if you’re not familiar with this type of surgery—we don’t do many thoracotomies here—and the instruments are unfamiliar, it becomes very challenging.” The contrast between P05’s proactive anticipation and P06’s struggle with unfamiliarity reveals that emergency response capability is a developmental continuum shaped by clinical exposure and deliberate practice. While novice nurses focus on reacting correctly to each instruction, experienced nurses develop a preconscious readiness that allows them to stay one step ahead of the surgical team’s needs.

### 4.2 Value and dilemmas of team collaboration

#### 4.2.1 Key supporting role of efficient collaboration

All interviewees acknowledged that efficient team collaboration is critical to the success of surgical management for patients with multiple and combined traumatic injuries. As these procedures involve multiple disciplines, clearly defined roles and mutual support among team members can reduce communication costs, avoid workflow disorganization, and improve treatment efficiency. Moreover, tacit coordination among medical staff is particularly important in emergency resuscitation, enabling seamless integration of various procedures and saving valuable treatment time for patients. Participant P02 stated: “Trauma resuscitation is a race against time. With clear division of labor and mutual backup, some of us handle instrument passing, others manage blood transfusion and fluid infusion, and others monitor vital signs. This tacit coordination can significantly shorten operative time and boost treatment success rates.” A more detailed account of team configuration during a complex triple-cavity surgery further illustrated this point: P11: “For a patient with severe trauma who needed simultaneous brain, chest, and abdominal surgery, we had five medical staff members—everyone had their own job. Two scrub nurses and three circulating nurses were responsible for blood transfusion, assisting the surgery, and all kinds of other tasks. Everyone stood their ground and cooperated closely. Without this kind of outstanding teamwork, it would have been absolutely impossible to complete such a complex and high-risk surgery.” This level of coordinated division of labor was seen as a direct driver of both surgical efficiency and patient outcomes: P06: “If there are enough of us off the table, you have one person specifically managing the anesthesia side for the patient’s bleeding, one person managing the count, and one helping the doctor gown up—this way, the surgery goes much faster. For the patient, shorter surgery time means better and faster post-operative recovery.”

P01 added: “If we can cooperate closely, the surgical treatment time will definitely be shortened, and the patient’s chance of successful treatment will be greater. Sometimes during the rescue, one person can’t pay attention to everything comprehensively—when team members remind each other in time, we can avoid causing additional harm to the patient.” Participant P04 further emphasized the cohesive force of a well-coordinated team: “A team with clear role allocation and tacit cooperation can quickly form a cohesive treatment force during emergencies, cut unnecessary delays, and improve patient outcomes.”

The participants’ accounts collectively highlight the clinical significance that nurses attribute to collaboration—it is not merely about workflow convenience, but fundamentally about patient survival and safety. From P02’s “race against time” to P11’s assertion that complex surgery would be “absolutely impossible” without teamwork, the centrality of collaboration to trauma care is unequivocal.

#### 4.2.2 Practical dilemmas in collaboration

Despite the clear value of team collaboration, cross-departmental and cross-team cooperation remains challenged by several practical dilemmas. First, communication conflicts may arise in high-pressure clinical settings. When surgeons, anesthesiologists, and nurses all perceive their own tasks as urgent, friction emerges:

Participant P04: “Because everyone is in this high-pressure environment—sometimes the doctor is in a hurry, the anesthesiologist feels that the anesthesia matters are urgent, the surgeon feels that the surgical matters are urgent, and you also feel that the things you’re doing are urgent. This leads to conflict and contradiction.”

P04’s triple repetition of “urgent” vividly conveys how competing urgencies undermine collaborative flow. Second, support response is often untimely. The long distance between the trauma center and the main operating room results in slow arrival of supporting staff during emergencies, leading to insufficient manpower for acute management: Participant P01: “It’s quite far from our central operating room. Sometimes when we’re overwhelmed and can’t manage, it’s not so convenient to call for help, and people don’t come that quickly.”

Third, information handover is incomplete. Gaps in the transmission of clinical information between the emergency department and the operating room may occur, and failure to convey critical details promptly and accurately increases the risk of coordination errors during surgery: Participant P01: “Sometimes when patients are transferred from the emergency department, clinical information is inadequately communicated. We have to recheck and verify the details, which delays preoperative preparation.” Beyond these structural and communication barriers, a fourth, often unspoken dilemma emerged: the emotional labor of absorbing negative emotions from multiple sources during teamwork, with no outlet for one’s own distress.

Participant P04: “Sometimes doctors may have negative emotions because the surgery isn’t going smoothly, and they’ll project that onto us nurses. We have to manage their emotions. When I have my own negative emotions from work, there’s nowhere to offload them—I can only bottle them up. It’s very painful.”

P04’s account reveals an important and underexplored dimension of teamwork: the emotional labor of being not only a collaborator but also an emotional buffer for the entire team. This creates a hidden psychological burden layered on top of the instrumental demands of surgical coordination—one that current team training and support structures do not adequately address.

### 4.3 Psychological stress and coping strategies

#### 4.3.1 Psychological responses in high-stress environments

Trauma operating room nurses routinely face high levels of psychological stress, which often presents as negative emotions, including anxiety, tension, and fear. A subset of these nurses are prone to developing compassion fatigue, stemming from prolonged exposure to high-stakes trauma resuscitation environments and repeated, direct contact with patients’ severe injuries and suffering. This psychological burden is further worsened by ongoing worries that surgical coordination errors could negatively affect patient outcomes. One nurse described the full psychological trajectory from acute anxiety to compassion fatigue: P12: “Critically injured trauma patients can deteriorate extremely rapidly, leaving us with almost no time to prepare. We must immediately and urgently deploy all instruments and consumables, while simultaneously worrying that this time might affect the patient’s rescue. This situation makes me extremely anxious. Sometimes, witnessing the severity of the patients’ traumatic injuries, I even feel fear, and I have experienced compassion fatigue.” P12’s account traces a psychological sequence: from acute anxiety about time pressure, to fear triggered by the visual reality of severe trauma, culminating in compassion fatigue—a cumulative emotional depletion. Other participants echoed this sense of overwhelming pressure in the face of rapidly changing conditions and insufficient resources: Participant P06: “The clinical status of patients with multiple injuries can shift in an instant, leaving us with almost no time to prepare. We have to quickly get all required instruments and supplies ready, while also feeling extremely anxious that coordination mistakes could put patient rescue efforts at risk. Sometimes, seeing patients’ severe traumatic injuries can also trigger intense fear and compassion fatigue.” Participant P02: “When multiple trauma surgeries run at the same time, understaffing leaves us spread far too thin. We face enormous psychological pressure, having to both guarantee the quality of surgical coordination and maintain patient safety.” The psychological burden is further compounded by the sensory and emotional impact of witnessing severe injuries: P04: “Every time I encounter trauma surgery, I feel like this patient is in such a terrible state, with wounds all over their body, everywhere needing treatment... Every time it feels very difficult, and my psychological pressure is quite high.” In the most extreme situations, the stress becomes existential, confronting nurses with the limits of their ability to save lives: P11: “It was a severe car accident polytrauma patient. We had to simultaneously open the chest, the skull, and the abdomen, with multiple departments performing resuscitation together. The patient went into multiple cardiac arrests during the surgery. The whole process was high-intensity resuscitation, and both the psychological and work pressure were especially enormous.”

P11’s experience illustrates how the immediacy of life-threatening events during surgery creates an acute psychological intensity that distinguishes trauma nursing from routine surgical care—a state where the boundary between professional composure and personal emotional overwhelm becomes critically thin.

#### 4.3.2 Self-adjustment and demand for external support

Over years of clinical practice, trauma operating room nurses have developed various psychological self-regulation strategies. These include proactively following up on patient information and discussing surgical plans with surgeons before operations to reduce stress through thorough preparation; exchanging experiences with colleagues and reviewing workflow gaps after operations to ease the psychological burden through reflection; and releasing negative emotions through physical exercise and emotional sharing. One nurse described a multi-phase approach encompassing proactive preparation, post-operative reflection, and peer exchange:

P01: “If it’s my on-call week and a TTA (Trauma Team Activation) is activated in the group chat, I’ll continuously monitor it. I keep watching the test results posted in the group, proactively communicate with the surgeon about the surgical plan before the procedure—this thorough preparation helps relieve stress. I also prepare for potential intraoperative emergencies. After the surgery, I’ll exchange and share experiences with colleagues, and review the shortcomings in our coordination process.”

Another nurse emphasized the importance of post-operative mental disengagement and physical release: P10: “During the surgery, I concentrate fully. After the surgery is over, I’ll find a quiet place, sit quietly for a while, go over the surgery process in my mind, and summarize what I did well and what needs improvement. After work, I try not to think about work stuff anymore—chat with family and friends, watch a favorite TV show, or go out and exercise, sweat a bit, to release all the stress.” Similarly, other participants described comparable strategies: Participant P05: “I proactively follow up on patient information from the trauma center, and discuss the surgical plan with surgeons in advance before the operation to get fully prepared and relieve stress. After the operation, I exchange and share experiences with colleagues, and review the shortcomings in our coordination process.” Alongside these independent self-regulation efforts, nurses also hold clear and urgent unmet needs for external support. In particular, they expect hospitals to provide timely staffing backup during emergencies, optimize work scheduling systems, and set up specialized psychological counseling services: P01: “but I still hope the hospital can provide professional psychological counseling services.” Participant P03: “We hope the hospital can optimize the scheduling system to avoid long periods of high-intensity work, and that we can get timely staffing support during emergencies to reduce work-related stress.”

Beyond these structural needs, the emotional toll of unexpressed distress was powerfully articulated:

P04: “I might talk to a colleague about it, or slowly try to regulate it myself. But when I have negative emotions at work, there’s nowhere to offload them—I can only bottle it up. It’s very painful.”

The juxtaposition of diverse self-regulation strategies (P01, P10, P05) with the explicit plea for professional psychological services (P01), the request for structural workplace improvements (P03), and the admission of suffering in silence with no emotional outlet (P04) reveals a critical finding: individual resilience alone is insufficient to sustain psychological wellbeing in this environment. The need for a systemic, institutional psychological support structure—one that does not depend on nurses’ own initiative to seek help—is both explicit and urgent.

### 4.4 Professional growth and support needs

#### 4.4.1 Enhancement of professional value

Through their involvement in trauma surgery and emergency resuscitation, nurses gain a strong sense of professional achievement and value recognition after successfully rescuing patients, and this serves as a key motivator for them to remain in trauma nursing positions long-term. All interviewed nurses shared that although coordinating trauma surgery is full of challenges, witnessing patients pull through from life-threatening conditions allows them to deeply feel the meaning of their work, and their professional identity grows stronger as a result. The moment of seeing a critically injured patient survive against overwhelming odds is particularly formative for professional identity: P04: “When you see a patient who was, you could say, on their last breath, or whose life was hanging by a thread, being pulled back and saved—I think that really gives you a certain sense of achievement.” P13: “It’s like snatching someone back from the brink of death, fighting with the King of Hell for a life. When the patient is safely transferred to ICU and then discharged, a life continues. This makes me feel that my work is extremely meaningful and valuable.” P13’s visceral metaphor of “fighting with the King of Hell” powerfully conveys the existential meaning that nurses derive from their work—a meaning that transcends conventional job satisfaction. Beyond the emotional reward of saving lives, professional growth itself was a source of value and pride: Participant P08: “Every time I successfully coordinate resuscitation for a critically ill trauma patient and watch them pull through, I can deeply feel the meaning and value of my work. This sense of achievement is the core motivation that keeps me going in this role.” Participant P01: “Repeatedly taking part in trauma surgery and resuscitation has not only improved my emergency management capabilities, but also deepened my understanding of nursing work, and my sense of professional value has kept increasing as a result.” Together, these accounts reveal that professional value is not merely an outcome of successful practice—it is a sustaining, generative force. Whether expressed as P08’s “core motivation,” P13’s existential battle against death, or P01’s cumulative growth in competence and understanding, the deep sense of meaning that nurses derive from trauma care enables them to continue working in a high-stress environment where material rewards may not be commensurate with the emotional and physical demands.

#### 4.4.2 Needs for improvement in training and work environment

In trauma surgical nursing, operating room nurses have clear and urgent needs for systematic specialized training and work environment optimization, which largely stem from the dilemmas they face in daily clinical work and the practical needs of their career development. These needs focus on four core areas.

First, staffing allocation optimization: nurses called for increased nursing manpower and flexible shift scheduling to relieve work pressure during peak surgical periods.

P01: “I think staffing should be increased, because multiple and combined traumas are severe and can change rapidly—the staffing ratio should definitely be different from elective surgery.” Second, emergency reserve improvement: nurses identified the need to supplement dedicated emergency supplies and establish a dynamic material management mechanism: P01: “Emergency surgical supplies should be increased, such as warming blankets, pressurized blood and fluid warmers, orthopedic tourniquets, because if you’re doing two surgeries simultaneously, these may not be enough.” Third, clinical workflow standardization: nurses highlighted the need for a standardized operating procedure (SOP) manual to provide clear work guidelines, particularly for newly recruited nurses: P02: “The trauma operating room has been running for quite a while now, and we have certain established processes. We can turn these processes into written SOPs, so that newly recruited and inexperienced staff have rules to follow when they encounter these situations, and won’t panic as much.”

Fourth, specialized skills training enhancement: nurses expressed strong preferences for practical, hands-on training over theory-dominated instruction: Participant P09: “We hope the hospital can increase specialized training for trauma surgery, especially simulation drills for multi-site combined surgery coordination and massive hemorrhage emergency management. At the same time, we expect more staffing, expanded emergency material reserves, and an improved SOP manual, so that newly recruited nurses have clear rules and guidelines to follow.” P09 also emphasized the need for an improved information exchange mechanism: “If everyone could do more simulation drills... or have a more efficient information exchange platform, that would be perfect.” Participant P04: “Current training is mostly theory-oriented and lacks practical drills. We hope to carry out scenario-based simulation training to improve our emergency coordination capabilities. I think we ultimately need more specialized training to improve the professional level of surgical staff, especially in orthopedics. “.

These requests collectively paint a portrait of a work environment where material resources are stretched, practical training opportunities are insufficient to meet the complex demands of trauma surgery, and institutional guidance—particularly for novice nurses—is not yet fully systematized. The nurses’ detailed and actionable suggestions, from specific supply items (warming blankets, pressure infusers, tourniquets) to simulation drills to SOP manuals, provide a concrete, evidence-based agenda for nursing management to address these identified gaps.

## 5 Discussion

### 5.1 Focus on core competency requirements to construct a specialized training system for trauma surgery

This study found that the professional challenges faced by operating room nurses in coordinating surgery for patients with multiple and combined traumatic injuries primarily stem from the inherent complexity, urgency, and high multidisciplinary collaboration demands of these procedures. This aligns with the findings of Shi et al. (21), who noted that trauma resuscitation places high demands on nurses’ professional competencies. Our study further clarifies the three core sources of these professional challenges within this specific clinical scenario. Most patients with multiple and combined traumatic injuries present with concurrent multi-site and multi-system injuries, which often leads to frequent shifts in surgical workflow. Compounded by the practical conflict between wound contamination and aseptic operation principles, as well as the unpredictability of intraoperative emergencies, these factors place more stringent clinical requirements on operating room nurses, in terms of professional knowledge reserve, emergency management capabilities, and operational precision. Deng et al. (22) noted that current trauma nursing education faces problems, including fragmented training content and limited clinical practice opportunities, and suggested strengthening systematic training for trauma specialist nurses, with a focus on integrating theory and practice. The specific competency requirements for nurses in trauma surgery identified in our study confirm the real-world impact of these training problems from the clinical practice perspective. Both findings point to the necessity and urgency of optimizing the specialized training system for trauma nursing. A recent concept analysis of trauma nursing competency by Baik et al. (15) provides a comprehensive international framework for this optimization, identifying six key attributes. The powerful firsthand accounts of our participants, such as the hyper-vigilance described by P15, extend this theoretical framework by revealing the real-world clinical manifestations of these competencies under extreme intraoperative pressure. Furthermore, the participants’ call for “scenario-based simulation training” (P09) echoes the findings of Murphy et al. (23), whose qualitative study demonstrated that simulation-based multidisciplinary Trauma Team Training improves non-technical skills and team performance. Based on these findings, we suggest that future efforts could develop an integrated three-component specialized training framework for trauma surgical nursing, combining theory, practical operations, and simulation. The theoretical training module could take the integration of multidisciplinary knowledge as its core, returning to the clinical essence of multi-system involvement and building a logical knowledge framework structured as “common trauma characteristics—inter-system correlation mechanisms—integrated nursing strategies”. Such a module would be intended to guide nurses in developing a holistic mindset for injury assessment and consolidate the theoretical foundation for comprehensive assessment capabilities. The practical operation training module would focus on high-frequency core clinical skills, emphasizing repeated drills and standardized implementation of key tasks, including surgical instrument management for multi-site procedures and emergency management of massive hemorrhage. This module would be designed to facilitate the direct translation of training content into clinical practice.

In addition, a multi-dimensional training effectiveness evaluation mechanism could be considered, adopting multiple assessment methods such as written theoretical tests, skill assessments, and scenario-based simulation evaluations. Such a mechanism would help nurses identify their own capability gaps and provide empirical feedback for nursing managers to optimize training programs. The potential scientific contribution of this proposed framework is that it provides preliminary experiential evidence bridging abstract competency frameworks (15) and context-specific training interventions for the intraoperative trauma setting, an area that remains understudied in the existing literature. In summary, an integrated three-component specialized training framework for trauma surgical nursing—covering theory, practical operation, and simulation—could potentially address the core competency needs identified in our study. However, this framework remains a suggestion based on our participants’ experiences; its effectiveness should be evaluated in future implementation research. **Readers are reminded that these suggestions derive from a single, high-volume tertiary trauma center; transferability to other settings (e.g., primary hospitals or different regional systems) requires careful assessment by readers based on contextual similarities.

### 5.2 Optimizing multidisciplinary collaboration mechanisms to improve coordination efficiency in trauma resuscitation

Our findings indicate that efficient team collaboration is key to improving the efficiency of trauma surgical treatment. However, in daily practice, cross-departmental collaboration still faces barriers, including poor information transmission, insufficient communication alignment, and delayed staffing support. These issues are particularly prominent in the linkage between the emergency department, surgical departments, the trauma operating room, and the main operating room, and they significantly affect overall treatment efficiency. This aligns with qualitative evidence that interdisciplinary communication barriers—such as perceived accessibility, cognitive biases, and hierarchy—can significantly affect trauma care outcomes (9). Existing studies have mostly focused on the construction and operation of multidisciplinary teams. In contrast, our study zooms in on the practical barriers to clinical collaboration, especially the linkage gaps between the emergency department and the operating room. The communication barriers identified by our participants resonate with but extend beyond the findings of Bankhead et al. (24), who revealed multiple forms of communication bias in trauma bays. Our participants’ accounts highlight additional structural and organizational sources of communication breakdowns, including spatial disconnect and the absence of standardized cross-departmental handover procedures. These converging findings suggest that optimizing trauma team communication requires a dual approach: addressing interpersonal biases through behavioral training and systemic barriers through organizational restructuring. Drawing on the mature trauma care collaboration experience from abroad (25) and relevant domestic research findings (26), and rooted in frontline clinical reality, we propose the following four potential strategies for improving collaboration, which should be tested in future practice:

First, we suggest establishing a triggered synchronous communication mechanism: via information systems or dedicated lines, the emergency department could alert the main operating room at the same time it notifies surgical departments, potentially reducing information delays and omissions.

Second, we propose implementing a standardized communication model: using the SBAR tool, a trauma patient handover checklist could be developed to standardize handover content, processes, and accountability, thereby reducing information errors caused by verbal communication.

Third, we recommend refining the multidisciplinary collaboration organizational structure: a specialized trauma resuscitation team could be established, with clarified responsibilities and collaboration processes, and continuous improvement could be achieved through regular debriefings.

Fourth, we advise optimizing the resource allocation and information sharing mechanism: through rational layout, dedicated emergency support channels, and an internal hospital information sharing platform, inter-departmental information barriers could be reduced, enabling the operating room to prepare in advance and respond rapidly.

Overall, these proposed optimization strategies address the existing problems in cross-departmental trauma surgery collaboration and carry potential practical value for improving treatment efficiency and quality. Future research should evaluate the feasibility and effectiveness of these strategies in real-world clinical settings. Readers should also note that these strategies reflect the organizational context of a single high-volume trauma center; their applicability may differ across institutions with varying resources, staffing models, or trauma system designs.

### 5.3 Building a diversified psychological support system to safeguard nurses’ professional mental health

Prior studies have established that nursing is a high-stress, high-demand professional field. However, existing research has notable gaps in exploring the complexity of workplace challenges specific to operating room nurses, as well as their differential impacts on nursing staff’s mental health (27). Our qualitative interviews revealed that operating room nurses coordinating surgery for multiple and combined trauma patients face a distinct constellation of stressors, including the urgency of surgery, unpredictability of patients’ conditions, sustained high workload, and the emotional labor of absorbing others’ negative emotions. The psychological toll identified in our study is not merely acute stress but a complex interplay of burnout, compassion fatigue, and secondary traumatic stress. The integrative review by Caulfield et al. (12) synthesized evidence on factors preceding occupational distress in emergency nurses, identifying that contributing factors arise from individual nurse characteristics, local organizational governance, and the inherent nature of the emergency nurse role. Our findings extend this framework into the operating room context, revealing that the intraoperative environment adds the unique dimension of being confined in a high-stakes space where the emotional labor of absorbing others’ negative emotions (P04) compounds pre-existing occupational distress factors. The scoping review by Tjugum et al. (8) of 36 studies specifically mapped the psychosocial factors influencing occupational stress in operating room nurses, identifying interprofessional challenges and high workload as primary contributors. Our data provide qualitative depth underpinning these quantitative findings, revealing the internal lived experience—such as feeling like a “jack-of-all-trades” who must manage everything simultaneously (P04). The mixed-methods study by Arnold et al. (28) identified compassion satisfaction as the strongest predictor of caring ability in emergency nurses, with most participants reporting low levels of compassion satisfaction. This finding is particularly significant in light of our participants’ accounts of professional achievement (P04, P13), suggesting that enhancing compassion satisfaction may serve as an important protective factor against psychological distress in the trauma operating room setting. Surendran et al. (10), in their qualitative exploration of emergency nurses’ cognitive mental workload, found that excessive cognitive demands were associated with burnout, compassion fatigue, and intention to leave. Our study extends these findings into the intraoperative context, revealing that the cognitive burden is further compounded by simultaneous multi-tasking across surgical fields and the constant emotional regulation required in a confined, high-pressure environment. While nurses in our study have developed individual self-regulation strategies (P01, P10), they explicitly stated a need for structured professional psychological counseling services, revealing a significant gap in institutional support. Our findings suggest that psychological wellbeing is not an individual responsibility but a systemic one. Based on these findings, we propose that a diversified psychological support system could be considered, integrating internal self-regulation support and external professional intervention, including dedicated psychological counseling services, peer support networks, workload optimization through flexible staffing, and normalized psychological early warning mechanisms. However, this proposal is derived solely from participants’ expressed needs; its feasibility, implementation, and effectiveness should be rigorously evaluated in future intervention studies.

Readers should also note that this proposed support system reflects the specific institutional context of a single high-volume tertiary trauma center; its applicability may vary across healthcare settings with different resource levels and organizational cultures.

### 5.4 Responding to nurses’ growth needs to optimize nursing management support strategies

Our findings indicate that successful participation in trauma surgical care grants nurses a strong sense of professional achievement, which serves as a key intrinsic motivator for them to remain in trauma nursing positions. This aligns with the findings of Gong et al. (29), who reported that nurses experience significant enhancements in professional identity and mission after managing trauma incidents during night shifts, alongside a clearer recognition of the meaning and value of nursing work. Together, these findings demonstrate that positive professional experiences effectively enhance nurses’ occupational stability, providing empirical evidence for building and retaining a stable trauma nursing workforce. Through qualitative interviews, this study further identified multiple practical barriers to the professional development of trauma nurses, including insufficient staffing, shortages of emergency supplies, poorly targeted specialized training, nonstandardized work procedures, and inadequate professional psychological support. These factors directly undermine nurses’ motivation for career advancement and represent key constraints in trauma nursing practice, highlighting the urgent need to optimize related management strategies. These multidimensional needs align with the organizational-level implications identified by Tjugum et al. (8), who emphasized in their scoping review that strong leadership, improved team dynamics, and supportive interventions are essential to mitigate occupational stress in operating room settings. Our study extends these implications by providing concrete, participant-driven recommendations grounded in frontline clinical experience.

Based on the findings of this study, targeted systematic management strategies are proposed to address the aforementioned challenges. These strategies should be considered as potential directions for future organizational improvement rather than empirically tested interventions. For staffing and resource support, the allocation of operating room nurses could be dynamically adjusted according to the complexity and workload of trauma surgeries, with priority given to recruiting core nurses experienced in assisting complex trauma operations. In addition, investment in emergency supply stockpiles might be increased, and a dynamic management and early warning mechanism for medical supplies could be established to ensure continuous and safe trauma care delivery. For workflow and system refinement, a standardized operating procedure (SOP) manual for trauma surgery coordination could be developed to standardize the entire nursing workflow, reduce operational deviations, and enhance both care quality and work efficiency. Additionally, the performance appraisal and incentive mechanism could be refined by incorporating metrics such as trauma surgery coordination competence and emergency response outcomes into evaluation criteria, alongside formal recognition and rewards for high-performing nurses. Management practices that integrate scientific rigor with humanistic care could further enhance nurses’ professional efficacy. For career growth support, a hierarchical nurse training system could be established, with personalized development plans tailored to the job demands of nurses at different seniority levels to help them systematically improve professional competence through diverse training modalities. Furthermore, a diversified career development platform might be built to support nurses’ engagement in trauma nursing-related research and teaching, fostering their growth into interdisciplinary talents. This approach could potentially address nurses’ career development needs while strengthening their professional identity and sense of belonging. The systematic management strategies proposed herein are closely aligned with the practical work demands and career development trajectories of trauma nurses. However, these suggestions are derived solely from participants’ lived experiences and require rigorous empirical evaluation in real-world clinical settings before widespread implementation. **Readers should also note that these management strategies reflect the specific human resource and organizational structure of a single high-volume tertiary trauma center; their applicability may differ across institutions with varying staffing ratios and administrative systems.

### 5.5 Strengths and limitations

Several limitations of this study need to be acknowledged. First, using purposive sampling, we recruited only 15 operating room nurses from a single tertiary hospital in Hunan Province, resulting in a geographically concentrated, homogeneous sample. The trauma care system, team collaboration model, and resource allocation at this study site may outperform those of primary care institutions, meaning our findings may not be generalizable to hospitals of other levels or regions. Future multicenter, large-sample studies are warranted to improve the generalizability of our conclusions.

Second, as a qualitative study, this research relies on researchers’ subjective interpretation. Although we implemented rigorous trustworthiness measures, including investigator triangulation, member checking, reflexive journaling, and external expert review, residual subjectivity remains unavoidable. Additionally, the depth and breadth of our semi-structured interviews were constrained by the interview guide and participants’ expressive capacity, which may have prevented us from fully exploring all potential themes. Follow-up research could integrate quantitative methods to mitigate subjective bias and enrich our findings.

Finally, this study did not conduct stratified analysis of contextual factors such as trauma surgery type and urgency level, which may have masked the heterogeneity of nurses’ experiences across different contexts. Future studies could incorporate more contextual variables and perform further stratified analysis to deepen the exploration of factors influencing trauma operating room nurses’ experiences.

It is also important to note that the study hospital is not an ordinary tertiary institution. It serves as a designated regional trauma center, a national key clinical construction specialist unit (General Surgery), and has received recognition from the National Center for Trauma Medicine. The center has established international collaborations (e.g., with Grady Memorial Hospital, USA) and provides trauma system guidance to multiple hospitals across provinces. Annually, it performs over 500 trauma surgeries covering orthopedics, thoracoabdominal, and neurosurgical trauma. Therefore, while the findings originate from a single center, the center itself is representative of high-volume, well-resourced tertiary trauma centers in China. Readers from similar settings may find the results more transferable than those from primary or low-volume institutions. However, transferability to other contexts should still be assessed by readers based on the similarity of their own clinical environments to the one described here.

Fourth, regarding the data collection period (January 2024 to July 2025, spanning 18 months), the extended timeline reflects both the iterative nature of qualitative sampling—continuing until data saturation was achieved (no new themes emerged from three consecutive interviews)—and logistical constraints. Specifically, in mid-2025, our trauma operating room underwent a planned system upgrade and temporary restructuring to meet international standards. During this transition, although basic trauma surgical services continued, the center recommended limiting new participant enrollment to avoid interference with clinical operations. Consequently, we completed data collection in July 2025 rather than extending further. No other contextual changes (e.g., staffing, institutional policies) occurred during this period that would systematically bias participants’ responses.

## 6 Conclusion

Using a phenomenological qualitative approach, this study systematically explored the lived experiences of operating room nurses during surgical management of severe multiple and combined trauma. We identified four core themes: Professional Challenges and Emergency Response, The Value and Dilemmas of Team Collaboration, Psychological Stress and Coping Strategies, and Career Growth and Support Needs. Our findings indicate that operating room nurses play a critical and multifaceted role in trauma care, where they must navigate concurrent pressures across three interconnected domains: professional skills, team collaboration, and psychological wellbeing. Their professional growth heavily relies on robust specialized training systems, efficient cross-departmental collaboration mechanisms, and consistent psychological support. Building on these empirical findings, nursing managers can tailor trauma surgery nursing management strategies accordingly: First, develop a specialized simulation-based training system focused on multiple and combined injuries, strengthening hands-on drills and multidisciplinary collaboration training to improve nurses’ emergency response capacity. Second, refine the multidisciplinary collaboration mechanism, establish standardized communication and handover protocols, and optimize resource allocation to boost team collaboration efficiency. Third, build a diversified psychological support system integrating internal self-regulation resources and external professional intervention, delivering regular psychological counseling and stress management training to alleviate nurses’ psychological burden. Fourth, attend to nurses’ career growth needs, optimize staffing and supply stockpiles, refine work procedures and incentive mechanisms, and strengthen their professional identity and resilience, ultimately improving the overall quality of trauma care and better aligning with the development demands of modern trauma nursing.

## Data Availability

The raw data supporting the conclusions of this article will be made available by the authors, without undue reservation.
